# Use of Guideline-Directed Medical Therapy in Patients With ST-Elevation Myocardial Infarction

**DOI:** 10.7759/cureus.9398

**Published:** 2020-07-26

**Authors:** Ahmad Nawid Latifi, Abeera Akram, Samir Dengle, Amjad Minhas, Carolina Borz-Baba

**Affiliations:** 1 Internal Medicine, Saint Mary's Hospital, Waterbury, USA; 2 Medicine, Saint Mary's Hospital, Waterbury, USA

**Keywords:** stemi, st-elevation myocardial infarction (stemi), dapt, guideline directed medical therapy

## Abstract

Introduction

ST-elevation myocardial infarction (STEMI) is a serious manifestation of coronary artery disease and remains a significant contributor to morbidity and mortality worldwide. To reduce the risk of recurrent cardiovascular disease (CVD) events, the American College of Cardiology (ACC) and American Heart Association (AHA) recommend the use of five classes of medications after acute coronary syndrome (ACS). The purpose of this study was to evaluate whether STEMI patients admitted to our community hospital were discharged on optimal medical therapy based on the latest AHA/ACC guidelines.

Methods

A retrospective, single-center electronic medical records review was conducted at our community hospital between July 2017 and December 2018. Patients included in the study were admitted to our hospital through the emergency department as STEMI alerts. We reviewed the discharge prescriptions and assessed compliance with the medication regimen endorsed by AHA/ACC, which includes aspirin, P2Y12 inhibitors, β-blockers, angiotensin-converting enzyme inhibitors (ACEIs) or angiotensin receptor blockers (ARBs), and statins.

Results

A total of 147 patients were included in our study. The mean age of our study population was 62 ± 12.48 years. 97.2% of all patients with STEMI underwent coronary angiography. Hypertension (65.9%) was the most common comorbidity followed by hyperlipidemia (54.42%), diabetes mellitus (29.25%), and history of coronary artery disease (CAD) (24.48%). Among patients with successful reperfusion, 87.4% of the patients received the combination of four guideline-directed medical therapy (GDMT) (comprising dual antiplatelet therapy, a β-blocker, and a statin) and 57% were discharged on five guideline-directed medical treatment (the combination of dual antiplatelet therapy, a β-blocker, an ACEIs or an ARB, and a statin).

Conclusion

Optimal secondary prevention medications are known to be effective in reducing the risk of repeat ischemic events in ACS. This study demonstrated that adherence to GDMT in our community-based hospital study is better compared to prior studies but remained suboptimal. Potential strategies to improve adherence to guidelines are necessary.

## Introduction

Coronary artery disease (CAD) is the leading cause of death in the USA [1]. Acute coronary syndrome (ACS) is classified into three different types: unstable angina (UA), non-ST-segment elevation myocardial infarction (NSTEMI), and ST-segment elevation MI (STEMI). Mortality rates at 30 days for patients presenting with ST-elevation myocardial infarction are between 2.5% to 10% [2]. STEMI is treated via percutaneous coronary intervention (PCI) which represents the principal reperfusion strategy. Continuing guideline-directed medical therapy after STEMI and PCI aims at preventing recurrences of ischemic cardiac events and improving morbidity and mortality of the patients discharged with a diagnosis of ACS. In this study, we reviewed the risk factors for CAD in our STEMI alert population, we identified the final diagnosis at the time of discharge, and we focused on compliance with guideline-directed medical therapy.

## Materials and methods

This was a retrospective, single-center study conducted in a community hospital. We reviewed the medical records of all the patients who presented as a STEMI alert to our emergency department from July 2017 until December 2018. The study included patients 18 years of age and older with persistent chest pain and ST-segment elevation ≥2 mm in two contiguous leads, criteria consistent with the third universal deﬁnition of myocardial infarction (type 1 myocardial infarction) [3]. Using electronic medical records (EMR), we collected baseline data including age, sex, presenting complaints, past medical history including hypertension, dyslipidemia, diabetes mellitus, coronary artery disease, smoking, and cocaine use. After coronary angiography, patients were divided into patients with confirmed CAD and patients without CAD. The discharge prescription for the patients with a diagnosis of CAD was reviewed using EMR.

The exclusion criteria included death during hospitalization, refusal of coronary angiography, or a diagnosis other than ACS after coronary angiography (e.g., Takotsubo syndrome, cocaine-induced vasospasm).

The protocol of the study was approved by the IRB committee. Informed consent was not required as the patient data were retrieved retrospectively, and the subjects were at no or low risk to breach their confidentiality and privacy.

Statistical analysis

Demographic variables, clinical characteristics, and discharge medications of patients selected in this study were examined using descriptive statistics. Frequencies (n) and percentages (%) were utilized to present categorical variables, whereas mean and standard deviation were used for continuous variables. The rates of complete versus incomplete prescription of guideline-directed medications at discharge were compared using the Chi-square test for equal proportions for categorical variables. All analyses were performed using SPSS version 25.0 (IBM Corp, Armonk, NY).

## Results

**Figure 1:**
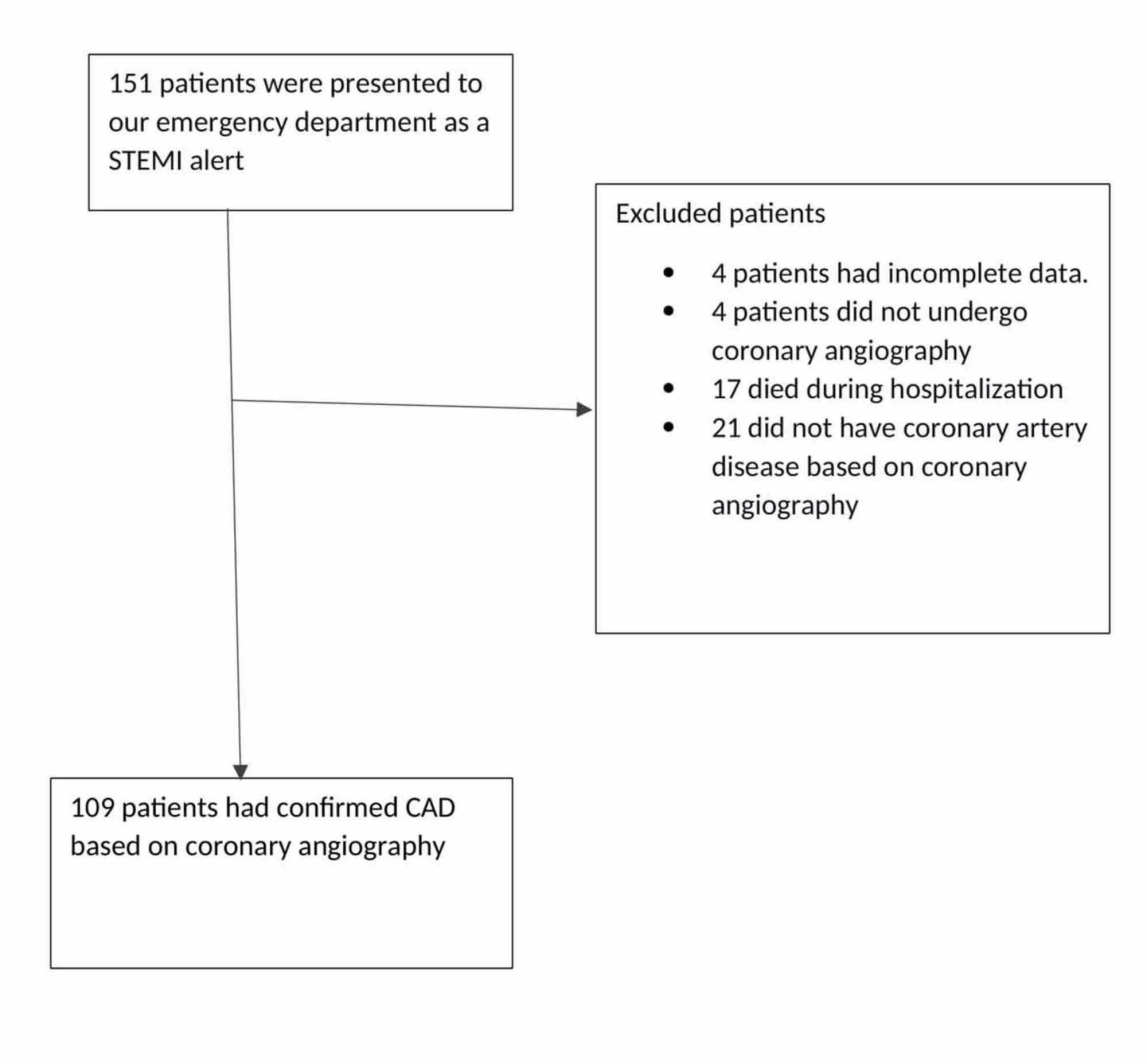
Patient distribution STEMI: ST-elevation myocardial infarction; CAD: Coronary artery disease.

A total of 151 patients presented to our emergency department as a STEMI alert during the study periods. Four patients were excluded due to incomplete data. One hundred forty-three or 97.27% of the remaining 147 patients with STEMI on presentation underwent coronary angiography. Four (2.72%) patients did not complete coronary angiography. One was deemed high risk, two were not consistent with a STEMI presentation per cardiology and one patient declined the procedure. We excluded patients who expired during hospitalization (11.5%) and those who did not have CAD based on coronary angiography (14.48%). Thus, the final number of patients included in our study was 109 (Figure 1).

The baseline characteristics of these patients are presented in Table 1. The majority of the patients were males 74 (70.06%) and 35 (29.94%) were females, with a mean age of 62.19 +/- 12.48 years. The main presenting complaint was chest pain (79.59%). Cardiac arrest was the presenting scenario in 5.44% of cases and a similar number of patients had only dyspnea on presentation. Altered mental status, syncope, dizziness, and stroke-like symptoms were the initial symptoms in the remaining patients (9.53%). Hypertension (65.9%) was the most common comorbidity among our subjects followed by hyperlipidemia (54.42%), diabetes mellitus (29.25%), and history of CAD (24.48%). History of cocaine use was recorded in 12.24% of the cases. Of 109 patients with confirmed CAD, 99 (91.3%) patients underwent PCI, nine (8.2%) had multi-vessel disease who were referred for coronary artery bypass grafting (CABG) and one patient was managed medically due to difficult anatomy. An ejection fraction (EF) of ≥50% was recorded in 62.3% based on echocardiography and 36.9% of patients had an EF ≤ 50%.

**Table 1:** Baseline characteristics of patients with confirmed coronary artery disease (n = 109) CABG: Coronary artery bypass grafting; PCI: Percutaneous coronary intervention.

Variables	Frequency (%)
Male	70.06%
Female	29.94%
Age	62.19 years +/- 12.48
Presenting complaint (n = 109)	
Chest pain	79.59%
Cardiac arrest	5.44%
Dyspnea	5.44%
Other (Altered mental status, syncope, dizziness and stroke-like symptoms)	9.53%
Cardiovascular risk factors (n = 109)	
Hypertension	65.9%
Hyperlipidemia	54.42%
Diabetes mellitus	29.25%
History of coronary artery disease	24.48%
History of cocaine abuse	12.24%
Therapeutic strategy (n = 109)	
PCI	91.3%
CABG	8.2%
Conservative treatment	0.9%
Ejection Fraction (n = 109)	
>/= 50%	62.3%
<50%	36.9%

**Figure 2:**
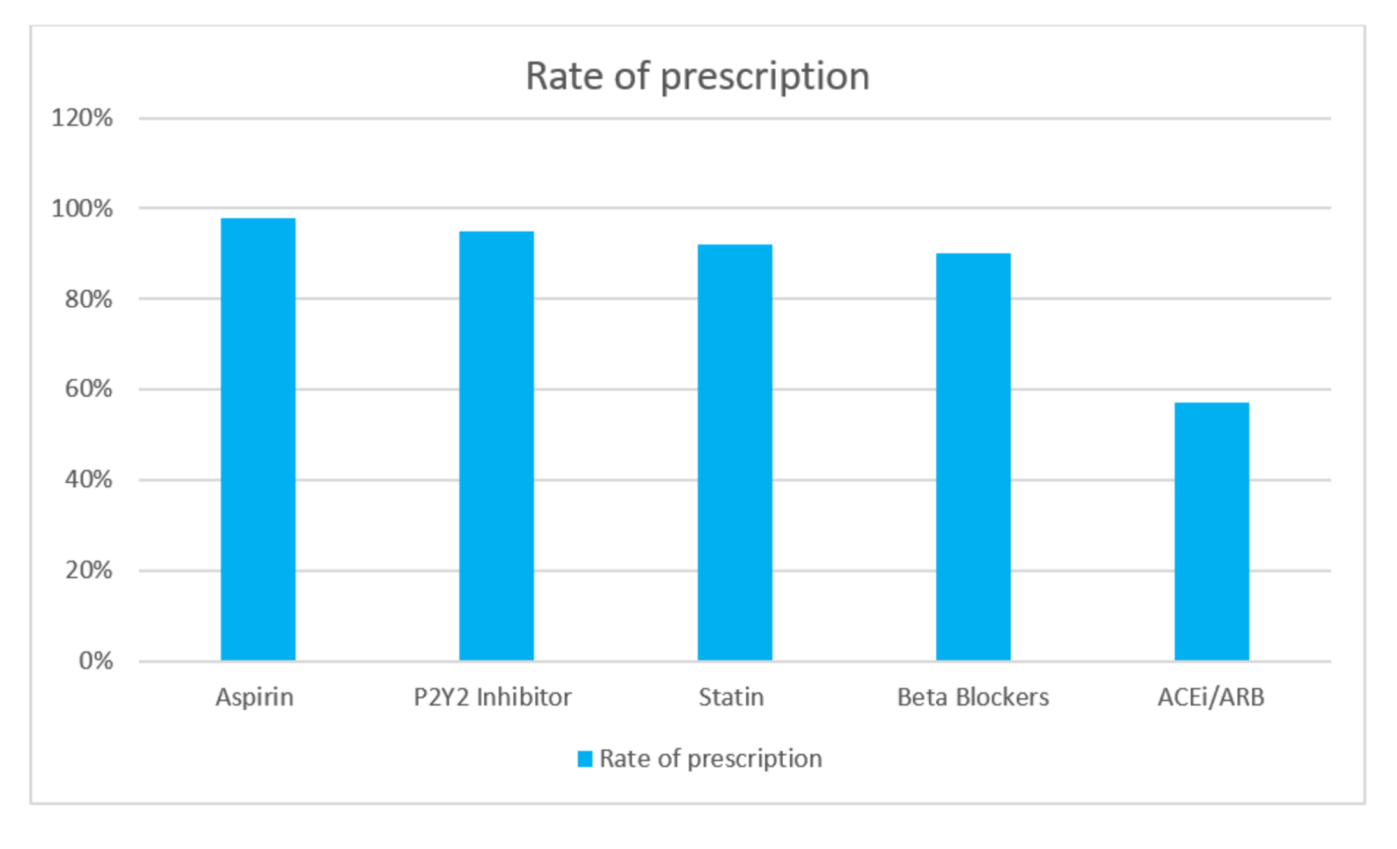
Rate of GDMT prescription on discharge GDMT: Guideline-directed medical therapy

Among patients with a confirmed diagnosis of CAD who underwent PCI, 97.9% were discharged on aspirin, 94.7% on second antiplatelet therapy (ticagrelor or clopidogrel), 90% on a β-blocker, 57% on an angiotensin-converting enzyme inhibitor (ACEI) or angiotensin receptor blocker (ARB) (71% with reduced EF), and 92% on a statin (Figure 2). Overall, 90% of the patients received the combination of four secondary prevention medications (including dual antiplatelet therapy, a β-blocker, and a statin) and 57% were discharged on five guidelines directed medical therapy (the combination of dual antiplatelet therapy, a β-blocker, an ACEIs or an ARB, and a statin). The rate of prescribing the five guideline-directed medical therapy (GDMT) varies based on risk factors as summarized in Table 2. The reasons for nonadherence to these five drug regimen are shown in Table 3.

**Table 2:** Percentage of patients received the Five Drug Therapy based on presence of risk factors and reduced LVEF LVEF: Left ventricle ejection fraction; CAD: Cardiovascular disease.

Risk Factors	Risk Factor +	Risk Factor -	P-value
Age <55 years	78%	46%	<0.001%
Male	49.3%	53%	0.248
Hypertension	60%	42%	<0.01
Diabetes mellitus	58%	52%	0.39
Hyperlipidemia	59%	49%	0.15
History of CAD	47%	45%	0.77
Smoker	50%	51%	0.88
Cocaine use	12%	54%	<0.0001
LVEF < 50%	63%	48%	0.03

**Table 3:** Reasons for not receiving recommended medication ACEI: Angiotensin-converting enzyme inhibitors; ARB: Angiotensin II receptor blocker; AKI: Acute kidney injury; EF: Ejection fraction.

S.No	Medications	% of patient not receiving it	Reason for not prescribing
1	Aspirin	2	High risk for bleeding
2	P2Y12 Inhibitors	5%	High risk for bleeding (4%), Not documented (1%)
3	Betablocker	10%	Bradycardia (3%), Hypotension (2%), Not documented (5%)
4	Statin	8%	Intolerant to statin/Myalgia (3%), Not documented (5%)
5	ACEI/ARB	43%	Preserved EF (20%), AKI (9%), Hypotension (7%), Not documented (7%)

## Discussion

Approximately 38% of patients who present to the hospital with the acute coronary syndrome have an ST-elevation myocardial infarction [4]. Early suspicion and prompt diagnosis of STEMI is crucial to patient management as early treatment with reperfusion significantly improves the outcomes [5]. Following acute coronary syndrome, patients have a high risk of subsequent ischemic events including myocardial infarction, stroke, and death. Thus, long-term secondary prevention strategies including optimal medical treatment and therapeutic lifestyle changes such as increased physical activity, dietary modification/weight loss, and smoking cessation are paramount and of proven benefit [6,7]. After the completion of early reperfusion treatment, the American Heart Association (AHA)/American College of Cardiology (ACC) recommend the long-term prescription of the combined drug regimens, including aspirin, P2Y12 inhibitor, β-blocker, statin, angiotensin-converting enzyme inhibitor (ACE-I) or angiotensin II receptor blocker (ARB) A [6].

Dual antiplatelet therapy (DAPT), β-blocker, and statin are class I recommendation per AHA/ACA guidelines. ACE I is also recommended strongly as class I in patients who are at high risk (i.e., anterior STEMI, heart failure, reduced EF) of subsequent cardiovascular events. In patients who are not at high risk, ACEI is reasonable for all patients with STEMI and no contraindications to their use (Class IIA, Level A) [6].

The rate of adherence to prescribing guideline-directed therapy among physicians is not optimal yet. A French study which collected data of more than 5000 STEMI patient between 2009 and 2013 showed that around 40% of STEMI patients were inadequately treated with at least one drug with a class I recommendation was missing [8].

Danchin et al. reported in 2000 that in the STEMI patient population, the optimal treatment combining antiplatelet therapy, statins, and β blockers was prescribed in 53% of individuals, and ACEI was initiated in only 29% of the patients upon discharge [9].

Adherence with secondary prevention therapy in patients with STEMI remains independently associated with lower one year mortality [8]. Bruggmann et al. demonstrated that a drug lacking at the time of discharge was rarely introduced within the year (lowering the chances of reducing future cardiovascular events) [10].

In our study, four out of the five guideline-directed medications were prescribed in 90% of cases, but only 57% of patients were discharged on five GDMT. This compares positively with compliance rates observed in the prior studies, owing to a strong collaboration between the prescriber and quality improvement.

The single important class of medication which has been less prescribed and the reason for the lower rate of five drug regimen is ACEI/ARB. The most common documented reason for not prescribing ACE I in these patients was preserved EF (20.1%). As mentioned earlier, although these patients (with preserved EF) are in the low-risk category for subsequent cardiovascular events, ACE I is encouraged to be given if there is no contraindication to reduce fatal and nonfatal major cardiovascular events [6].

STEMI in patients younger than 55 seems to be associated with a statistically significant higher rate (p-value < 0.001) of compliance with guidelines. The presence of hypertension indicates the potential need to add an ACEI/ARB which increases adherence as well (p-value < 0.01). An EF ≤ 50% was correlated with a statistically significant (p-value < 0.03) improvement in prescribing five drug regimen prescriptions. Cocaine use was associated with reluctancy to start a five drug regimen (P-value < 0.0001). There was no significant difference between gender and other comorbidities in regard to the rate of optimal medical therapy (Table 3).

These results are encouraging but confer us with the opportunity to reflect on strategies to further improve the prescription rate of secondary prevention drugs. Implementation of a standardized prescription order set in EMR based on the latest guideline could reduce the proportion of suboptimal prescriptions at discharge. These standardized prescriptions can be applied by a pharmacist or a nurse run program. Patient education and frequent outpatient follow-up after the initiation of this medication regimen should be considered in patients who experienced complications secondary to medication such as hypotension and bradycardia which could be either transient or to a certain degree expected, as worsening creatinine after the introduction of ACEI/ARBs.

Our study has limitations that should be mentioned. The most important is the small study population and the retrospective type of analysis which provides only descriptive data. All data were collected through the review of electronic medical records. Consequently, we may have underestimated the prescription rates for the drugs studied.

## Conclusions

In our study, the majority of the patients with a diagnosis of STEMI received medical therapy with four classes of guideline-directed therapy and more than half of these patients were prescribed all five optimal medical therapy. Age younger than 55, hypertension (HTN), and reduced EF associated with a higher rate of all five classes of GDMT. Our study shows overall a better adherence to guideline-directed medical therapy compared to other studies. Nonetheless, the rate of GDMT prescription on discharge in these vulnerable patients who are at much higher risk for recurrence ischemic events was suboptimal. Hospital discharge offers a good opportunity for improvement of quality of care, therefore strategies should be implemented at the time of discharge to increase compliance to well-validated guidelines.
